# Comparison of Long-Term Outcomes of Endoscopic and Minimally Invasive Catheter Evacuation for the Treatment of Spontaneous Cerebellar Hemorrhage

**DOI:** 10.1007/s12975-020-00827-8

**Published:** 2020-07-04

**Authors:** Leiyang Li, Haixiao Liu, Jianing Luo, Zhijun Tan, Junmei Gao, Ping Wang, Wenting Jing, Ruixi Fan, Xiaoyang Zhang, Hao Guo, Hao Bai, Wenxing Cui, Xun Wu, Yan Qu, Wei Guo

**Affiliations:** 1 Department of Neurosurgery, Tangdu Hospital, the Fourth Military Medical University, Xi’an, Shaanxi China; 2 Institute of Basic Medical, the Fourth Military Medical University, Xi’an, Shaanxi China; 3 Department of Health Statistics, the Fourth Military Medical University, Xi’an, Shaanxi China

**Keywords:** Spontaneous cerebellar hemorrhage, Neuroendoscopy, Minimally invasive catheter, Retrospective studies, Treatment outcome

## Abstract

Recently, minimally invasive techniques, including endoscopic evacuation and minimally invasive catheter (MIC) evacuation, have been used for the treatment of patients with spontaneous cerebellar hemorrhage (SCH). However, credible evidence is still needed to validate the effects of these techniques. To explore the long-term outcomes of both surgical techniques in the treatment of SCH. Fifty-two patients with SCH who received endoscopic evacuation or MIC evacuation were retrospectively reviewed. Six-month mortality and the modified Rankin Scale (mRS) score were the primary and secondary outcomes, respectively. A multivariate logistic regression model was used to assess the effects of the different surgical techniques on patient outcomes. In the present study, the mortality rate for the entire cohort was 34.6%. Univariate analysis showed that the surgical technique and preoperative Glasgow Coma Scale (GCS) score affected 6-month mortality. However, no variables were found to be correlated with 6-month mRS scores. Further multivariate analysis demonstrated that 6-month mortality in the endoscopic evacuation group was significantly lower than that in the MIC evacuation group (OR = 4.346, 95% CI 1.056 to 17.886). The 6-month mortality rate in the preoperative GCS 9–14 group was significantly lower than that in the GCS 3–8 group (OR = 7.328, 95% CI 1.723 to 31.170). Compared with MIC evacuation, endoscopic evacuation significantly decreased 6-month mortality in SCH patients. These preliminary results warrant further large, prospective, randomized studies.

## Introduction

Spontaneous cerebellar hemorrhage (SCH), which is the least common type of intracranial hemorrhage (ICH), accounts for approximately 9 to 10% of all ICH cases [1, 2]. The reported mortality rates vary widely, ranging from 20 to 75%, perhaps due to different treatment strategies [3].

Since the posterior fossa is a narrow space with no additional room for expansion, obstructive hydrocephalus, brainstem compression, and cerebellar herniation arising from the mass effect of hematoma and perilesional edema are major risk factors for a rapidly worsening prognosis in the acute stage [4, 5]. More importantly, previous studies have demonstrated that early surgical intervention can decrease the mortality rate in SCH patients associated with brainstem compression or hydrocephalus or with a hematoma > 3 cm in diameter [6–8].

However, suboccipital craniectomy has been the mainstay surgical intervention for SCH in the past [9, 10]. In recent years, with the development of less invasive techniques, minimally invasive approaches, including endoscopic evacuation and minimally invasive catheter (MIC) evacuation, have been widely used in clinical practice [11, 12].

To date, no clinical studies have compared the long-term outcomes of endoscopic evacuation and MIC evacuation in SCH patients. We conducted a retrospective study to analyze and compare the therapeutic effects of these 2 surgical techniques in the treatment of SCH.

## Methods

### Study Design and Patients

The present study was designed to compare the 6-month clinical outcomes of spontaneous cerebellar hematoma (SCH) patients who received either MIC evacuation or endoscopic evacuation within 48 h after onset. The protocol for this retrospective study was approved by the Biological and Medical Ethics Committee of Tangdu Hospital (No. TDLL–2014115). The research was performed strictly following the Declaration of Helsinki (2013) principles, and informed consent was waived by the Biological and Medical Ethics Committee.

Medical records of all cerebellar hematoma patients in the Department of Neurosurgery of Tangdu Hospital from July 1, 2015, to June 31, 2018, were reviewed. Then, the patients were screened according to the following selection criteria.

#### Inclusion Criteria

The age of the patients was ≥ 18 years.

Cerebellar hemorrhage was confirmed by computed tomography (CT) scans.

The hematoma was > 3 cm in diameter.

The patients underwent endoscopic evacuation or MIC evacuation within 48 h of ictus.

#### Exclusion Criteria

Hemorrhage was caused by secondary factors (trauma, neoplasm, aneurysm, arteriovenous malformation, hemorrhagic transformation of ischemic infarction, cerebral amyloid angiopathy).

There were multiple cerebellar hemorrhages or concurrent intracerebral hemorrhages.

The patients had known advanced dementia or disability before onset.

The patients had severe organ (cardiac, hepatic, renal, or pulmonary) dysfunction before onset.

The patients were confirmed to have malignant disease or a life expectancy of less than 6 months due to comorbidities before onset.

There was a concurrent serious infectious disease (HIV, tuberculosis, etc.).

The patients had concurrent coagulation disorders or were using antiplatelet or anticoagulant drugs.

The patients were pregnant or lactating.

The patients or relatives refused follow-up.

### Interventions

Patients were diagnosed according to the guidelines for the primary prevention of stroke [13]. All patients received standard medical treatment and care according to the recommendations in the guidelines of the American Heart Association/American Stroke Association (AHA/ASA) and European Stroke Organization (ESO) [14, 15]. External ventricular drainage was performed in patients with obstructive hydrocephalus. Before surgery, patients and their relatives were briefed on the surgical approach used for MIC evacuation or endoscopic evacuation. All surgeries were conducted by a well-trained surgical team.

### MIC Evacuation

A skull CT scan was performed to determine the location of the hematoma. After that, the drainage tube was implanted into the hematoma cavity along its main axis, avoiding damaging the venous sinus, brainstem, and important areas of the cortex. After the target was successfully introduced into the drainage tube, the hematoma was slowly aspirated with a 5 ml syringe.

A CT scan was performed postoperatively to ensure that the drainage tube was located in the center of the hematoma. Twenty thousand units of urokinase dissolved in 2 ml of normal saline were injected into the center of the cavity through the drainage tube. Then, the drainage tube was clamped for 4 h before it was reopened. The CT scans were reperformed every 24 h after surgery to evaluate the amount of residual hematoma and to determine when the drainage tube should be removed.

### Endoscopic Evacuation

A bone flap with a diameter of 1 cm to 1.5 cm was made on the skull under the principle of a close location to the hematoma center and avoiding damage to the venous sinus, brainstem, and important areas of the cortex. An appropriate incision was made over the epidural layer, and a puncture cannula was used to construct a minimally invasive surgical channel. The endoscope sheath was placed approximately two-thirds of the way to the hematoma’s distal margin parallel to its long axis. Upon achieving hemostasis, the endoscope sheath was retracted so that it was one-third of the way into the hematoma cavity. A coagulation suction tube (Karl Storz, Tuttlingen, Germany) was used to aspirate the hematoma by smooth-tip suction. The deep part of the hematoma was removed first. Along with the sheath withdrawn, the residual part of the hematoma was gushed out through the sheath. Repetitive irrigation and aspiration were performed to ensure satisfactory evacuation of the hematoma. In the event of intraoperative bleeding refractory to irrigation, monopolar cauterization was advanced through the coagulation suction tube. The endoscope was introduced to observe the residual space to ensure that the hematoma was removed and that there was no ongoing bleeding. The dura and skin were closed in a routine manner.

### Data Collection

All of the data in the present study were collected by researchers who were blinded to the purpose and design. The consecutive SCH patient list was provided by the Patient Information Management Department of Tangdu Hospital. The patients’ basic information, disease history, and treatment information were collected from the in-patient medical record system. The prognostic information was collected via telephone or by using WeChat (an instant messaging and social media APP) during follow-up.

The hematoma volume was calculated from CT scans using the formula A*B*C/2, where *A* was the greatest diameter on the largest hemorrhage slice, *B* was the maximal diameter perpendicular to *A*, and *C* was the vertical hematoma depth. For all the patients, preoperative and postoperative perihematomal edemas (PHEs) were determined with 3D Slicer software. Additionally, the PHE at the time of catheter removal in MIC group was also determined. The measurements were performed by outlining the rim of PHE on axial slices with a semiautomated edge detection tool. After inspection, the boundaries could be adjusted in the software’s editing module, which allows for better assessment.

### Outcome Evaluation

The primary outcome was the 6-month mortality rate. The secondary outcome was the long-term neurological functional status, evaluated by the modified Rankin Scale (mRS) score at 6 months. Adverse events, including reoperation, rebleeding, hydrocephalus, intracranial infection, and pulmonary infection, were also recorded.

### Statistical Analysis

Statistical analysis was conducted by statisticians who were blinded to the study’s purpose and design. Given the selection bias inherent to retrospective observational studies, the chi-square test was used to test the intergroup balance of the possible confounding factor. A *P* value of < 0.1 was considered unbalanced. The univariate analysis and the multivariate logistic regression model were used to analyze the independent effect of the surgical technique on outcome.

The mRS score was dichotomized as either a poor outcome (mRS score of 3–6) or a favorable outcome (mRS score of 0–2). A chi-square test was used to analyze the categorical variables. The variables are expressed as the mean ± SD. Statistical significance was assumed with a probability value of < 0.05. All the analyses were conducted using SAS/STAT version 9.4.

## Results

### Patient Numbers

A total of 192 SCH patients admitted to the Department of Neurosurgery of Tangdu Hospital from July 2015 to June 2018 were retrospectively reviewed. Ultimately, 52 consecutive patients (27 males and 25 females) were enrolled in the present study according to the selection criteria: there were 27 endoscopic evacuation patients and 25 MIC evacuation patients. All patients were Chinese Hans.

### Basic Characteristics

The statistical features of the data are shown briefly (Table 1 and Table 2). Twenty-five and 27 patients were enrolled in the MIC evacuation and endoscopic evacuation groups, respectively. The median age was 68 years with a range from 43 to 76 years in the MIC evacuation group and 66 years with a range from 40 to 80 years in the endoscopic evacuation group. The mean preoperative GCS scores in the MIC evacuation and endoscopic evacuation groups were 9.00 ± 3.07 and 10.11 ± 2.56, respectively. The mean interval between onset and operation was 17.19 ± 7.79 h in the MIC groups and 23.03 ± 23.83 h in the endoscopic evacuation group. The mean preoperative hematoma volumes were 21.56 ± 8.64 ml in the MIC evacuation group and 21.91 ± 7.21 ml in the endoscopic evacuation group (Table 1). The mean postoperative hematoma volume was 11.13 ± 4.05 ml in MIC group and 2.74 ± 2.63 ml in the endoscopic group. The mean evacuation rate after surgery was 44.1% ± 17.7% in MIC group and 86.7% ± 11.8% in endoscopic group. The mean residual hematoma volume was 4.30 ± 2.96 ml before the catheter removed in MIC group. The mean preoperative perihematomal edema (PHE) was 9.96 ± 5.60 ml in MIC group and 9.93 ± 8.10 ml in the endoscopic group. The mean postoperative PHE was 18.09 ± 11.23 ml in MIC group and 22.48 ± 11.76 ml in the endoscopic group. At the time of catheter removal in MIC group, the mean PHE was 26.87 ± 10.81 ml.

**Table 1:** Statistical description of the enrolled sample

Characteristic	Surgical methods (in whole patients) (*n* = 52)							
	Stereotactic aspiration				Endoscopic evacuation			
	*N*	Median	Mean	SD	*N*	Median	Mean	SD
Age (years)	25	68	65.72	8	27	66	63.63	10.91
Initial GCS score	25	8	9	3.07	27	10	10.11	2.56
Interval between onset and operation (h)	25	15.73	17.19	7.79	27	14.01	23.03	23.83
Hematoma volume (ml)	25	20.00	21.56	8.64	27	20.00	21.91	7.21

*n* number of cases, *SD* standard deviation, *GCS* Glasgow Coma Scale

**Table 2:** Statistical description of the survived cases

Characteristic	Surgical methods (in survivors) (*n* = 34)							
	Stereotactic aspiration				Endoscopic evacuation			
	*N*	Median	Mean	SD	*N*	Median	Mean	SD
Age (years)	11	66	62.45	8.98	23	66	63.35	11.72
Initial GCS score	11	10	10.27	3.38	23	10	10.48	2.39
Interval between onset and operation (h)	11	17.00	19.64	9.08	23	14.01	22.82	24.29
Hematoma volume (ml)	11	19.00	20.09	7.73	23	19.60	21.06	6.91

*n* number of cases, *SD* standard deviation, *GCS* Glasgow Coma Scale

In the present study, the mean length of ICU stay in the MIC group was 7.32 ± 4.33 days and 6.81 ± 4.85 days in the endoscopic evacuation group. In the MIC group, the mean length of ICU stay for preoperative GCS ≤ 8 patients was 8.21 ± 4.32 days and 6.18 ± 4.26 days for preoperative GCS> 8 patients. In the endoscopic group, the mean length of ICU stay for preoperative GCS ≤ 8 patients was 8.50 ± 3.37 days and 5.80 ± 5.39 days for preoperative GCS> 8 patients. Among the surviving patients, the mean length of ICU stay in MIC group was 5.27 ± 2.10 days and 6.43 ± 4.73 days in the endoscopic group.

A total of 11 patients from the MIC group and 23 patients from the endoscopic evacuation group survived 6 months after surgery. Among the survivors, the median age was 66 years with a range from 43 to 73 years in the MIC evacuation group and 66 years with a range from 40 to 79 years in the endoscopic evacuation group. The mean preoperative GCS scores in the MIC evacuation and endoscopic evacuation groups were 10.27 ± 3.38 and 10.48 ± 2.39, respectively. The mean interval between onset and operation was 19.64 ± 9.08 h in the MIC group and 22.82 ± 24.29 h in the endoscopic evacuation group. The mean preoperative hematoma volume was 20.09 ± 7.73 ml in the MIC evacuation group and 21.06 ± 6.91 ml in the endoscopic evacuation group (Table 2).

The variables of sex, age, preoperative GCS score, interval between onset and operation, and decompressive craniectomy were involved in the intergroup equilibrium analysis. The results showed that all of these variables were not significantly different between the MIC evacuation and endoscopic evacuation groups in either the whole cohort or the survivors (Table 3).

**Table 3:** The result of inter-group equilibrium analysis

Characteristic		Surgical methods (in whole patients) (*n* = 52)				Surgical methods (in survivors) (*n* = 34)			
		Total	Stereotactic aspiration	Endoscopic evacuation	*P* value	Total	Stereotactic aspiration	Endoscopic evacuation	*P* value
Gender	Male	27	11	16	0.2712	18	4	14	0.1805
	Female	25	14	11		16	7	9	
Age (years)	< 60	14	6	8	0.6475	12	4	8	0.9281
	> = 60	38	19	19		22	7	15	
Initial GCS score	3–8	24	14	10	0.1705	11	4	7	0.7296
	9–14	28	11	17		23	7	16	
Interval between onset and operation (h)	< 24	42	22	20	0.2030	27	9	18	0.8103
	≥ 24	10	3	7		7	2	5	
Decompressive craniectomy	Yes	1	0	1	0.3312	1	0	1	0.4827
	No	51	25	26		33	11	22	

*n* number of cases, *GCS* Glasgow Coma Scale

### Outcome Assessment

The 6-month mortality rates in the MIC evacuation group and endoscopic evacuation group were 56.0% (14/25) and 14.8% (4/27), respectively. In MIC group, 2 patients who were deeply comatose on admission did not improve postoperatively and died shortly after. One patient who experienced rebleeding died shortly after the emergency decompressive craniotomy. Four patients did not improve postoperatively and had withdrawal of life support. Seven patients had related medical complications and died in the long-term care facility. In endoscopic group, 2 patients experienced neurological deterioration after endoscopic evacuation and then underwent decompressive craniotomy. One of them died shortly after the reoperation. Two patients did not improve post endoscopic evacuation and then had withdrawal of life support. One patient died in the long-term care facility.

Among the survivors, 81.8% (9/11) of MIC evacuation patients and 71.9% (17/23) of endoscopic evacuation patients achieved good neurological function (mRS score ≤ 2) 6 months after surgery. There were 3 adverse events (2 cases of rebleeding and 1 case of reoperation) in the MIC evacuation group and 4 adverse events (2 cases of pulmonary infection and 2 cases of reoperation) in the endoscopic evacuation group.

The univariate analysis was conducted to assess the long-term outcomes after surgery and to determine which variables should be involved in the multivariate analysis model. The mortality in the endoscopic evacuation group was significantly lower (*P* = 0.0018) than that in the MIC evacuation group. In addition, the patients with an initial GCS score ≤ 8 had a higher mortality rate (*P* = 0.0061) than those with an initial GCS score ≥ 9. However, among the surviving patients, no variables were found to be correlated with the 6-month mRS score (Table 4).

**Table 4:** Results of univariate analysis about the influence of the involved factors on outcomes at 6 month

Characteristic		Mortality (*n* = 52)				mRS score (*n* = 34)			
		Total	Die	Survive	*P* Value	Total	3–5	0–2	*P* Value
Surgical methods	Stereotactic aspiration	25	14	11	0.0018*	11	2	9	0.6112
	Endoscopic evacuation	27	4	23		23	6	17	
Gender	Male	27	9	18	0.8400	18	6	12	0.1529
	Female	25	9	16		16	2	14	
Age (years)	< 60	14	2	12	0.0614	12	2	10	0.4860
	> = 60	38	16	22		22	6	16	
Initial GCS score	3–8	24	13	11	0.0061*	11	4	7	0.2224
	9–14	28	5	23		23	4	19	
Interval between onset and operation (h)	< 24	42	15	27	0.7328	27	7	20	0.5176
	≥ 24	10	3	7		7	1	6	
Decompressive craniectomy	Yes	1	0	1	0.4625	1	0	1	0.5734
	No	51	18	33		33	8	25	

*n* number of cases, *mRS* modified Rankin Scale, *GCS* Glasgow Coma Scale. *P < 0.05

Bold data: Univariate analysis showed that the mortality in the endoscopic evacuation group was significantly lower than the MIC group (P = 0.0018). The patients with GCS score ≤ 8 had a significantly higher mortality rate than those with GCS score ≥ 9 (P = 0.0061)

The multivariate logistic regression model was used to analyze the effect of the surgical approach on mortality rate (Table 5). The risk of death in the MIC evacuation group was 4.346 times (95% CI 1.056 to 17.886) that in the endoscopic evacuation group. In addition, the risk of death in patients with a preoperative GCS score ≤ 8 was 7.328 times (95% CI 1.723 to 31.170) that in patients with a preoperative GCS score ≥ 9.

**Table 5:** The result of multivariate logistic regression model analyses

Characteristic		Mortality (*n* = 52)		mRS score (*n* = 34)	
		OR	95% CI	OR	95% CI
Surgical methods (reference is endoscopic evacuation)	Stereotactic aspiration	4.346	(1.056, 17.886)	–	–
Initial GCS score (reference is 9–14)	3–8	7.328	(1.723, 31.170)	–	–

*n* number of cases, *mRS* modified Rankin Scale, *GCS* Glasgow Coma Scale

## Discussion

Suboccipital craniectomy has been the main surgical intervention used in the past. However, with the development of more advanced techniques, minimally invasive techniques have shown good therapeutic effects for the treatment of SCH patients [11, 16–18]. In the present study, the overall 6-month mortality rate was 34.6% (18/52), which was within the reported range of mortality rates between 20 and 50% in the literature [19]. However, after subdividing the patients into different treatment groups, there was a significantly higher mortality rate in the MIC evacuation group of 56.0% (14/25) compared with the mortality rate of 14.8% (4/27) in the endoscopic evacuation group.

Previous studies have reported that MIC evacuation can be a safe and effective treatment option for SCH patients. For example, Mohadjer et al. [16] reported a mortality rate of 7.1% (1/14) after 6 months, and Lee et al. [11] reported a long-term mortality rate of 3.8% (1/26). However, this low mortality rate could be explained by the smaller volume of the hematoma and the better neurological status upon admission. Our results demonstrate that when SCH patients have a large hematoma volume and worse neurological status upon admission, MIC evacuation is not the ideal choice.

We previously reported that compared with MIC evacuation, endoscopic evacuation could significantly decrease 6-month mortality in patients with basal ganglia hemorrhage [20]. However, among the surviving patients, only 22.4% achieved a good neurological recovery (mRS score ≤ 2) at 6 months. Unlike the poor neurological prognosis of basal ganglia hemorrhage, the majority of SCH patients who do survive subsequently experience a good neurological outcome with the ability to function independently [21]. In the present study, 76.5% (26/34) of the patients had a good neurological recovery (mRS score ≤ 2) at 6 months. The data described above demonstrated that once the SCH patients had survived the acute phase, they had a high probability of achieving a good neurological recovery. Thus, exploring the optimal surgical strategy is particularly important for these patients.

To our knowledge, evidence supporting endoscopic evacuation for SCH patients is lacking. Recently, Khattar et al. reported the outcomes of endoscopic evacuation for 6 SCH patients with Apollo or Artemis Neuro Evacuation Devices [18]. The mortality rate in their study was 33.3% (2/6), and no operative complications were recorded. More intriguingly, a much lower mortality rate (14.8%) was found in our cohort, and only 2 patients required subsequent craniectomy. These studies demonstrated that endoscopic evacuation could represent a reasonable alternative to conventional surgery for the treatment of SCH patients. However, further studies are needed to clarify the perioperative risk to benefit ratios for this technique.

The impaired consciousness observed in SCH patients can be largely attributed to the development of hydrocephalus and/or direct brain stem compression [22, 23]. Accumulating evidence has demonstrated that a low preoperative GCS score is a strong risk factor for poor prognosis after SCH [19, 24]. Wu et al. [25] demonstrated that a preoperative GCS score ≤ 8 was a strong predictor of mortality, reporting that SCH patients with GCS scores ≤ 8 were approximately 32 times more likely to die within the initial 7 days. Chang et al. [5] showed a significant correlation between 6-month mortality and GCS scores ≤ 8. In agreement with these studies, the present study found that a low GCS score (≤ 8) was a predictor of mortality 6 months after SCH. Notably, after subdividing the patients with preoperative GCS scores ≤ 8 into different treatment groups, the mortality rate in the endoscopic evacuation group was 30.0% (3/10), whereas it was 71.4% (10/14) in the MIC evacuation group. This preliminary result indicates that endoscopic evacuation might even decrease the mortality of preoperative comatose SCH patients. However, these results still need to be thoroughly tested and confirmed in a larger cohort.

This study has several limitations. First, owing to the retrospective nature of the study, the possibility of selection bias cannot be excluded, and some data were not uniformly ascertained. Second, potential methodological limitations might have influenced the results. Third, this study was carried out at a single center and may have limited generalizability. Multicenter randomized controlled trials are needed to confirm the effectiveness of different surgical techniques.

## Conclusion

In our cohort, compared with MIC evacuation, endoscopic evacuation significantly decreased the 6-month mortality of SCH patients. These preliminary results warrant further large, prospective, randomized studies.
